# Effect of blood pressure and total cholesterol measurement on risk prediction using the Systematic COronary Risk Evaluation (SCORE)

**DOI:** 10.1186/s12872-018-0823-3

**Published:** 2018-05-04

**Authors:** Sabina Ulbricht, Stefan Gross, Eva Brammen, Franziska Weymar, Ulrich John, Christian Meyer, Marcus Dörr

**Affiliations:** 1 University Medicine Greifswald, Institute of Social Medicine and Prevention, Greifswald, Walther-Rathenau-Str. 48, 17475 Greifswald, Germany; 2 German Centre for Cardiovascular Research (DZHK), Partner Site Greifswald, Fleischmannstr. 42-44, 17475 Greifswald, Germany; 3 Department of Internal Medicine B, University Medicine Greifswald, Ferdinand-Sauerbruch-Str, 17475 Greifswald, Germany; 4 University Medicine Greifswald, Institute for Community Medicine, Section Epidemiology of Health Care and Community Health, Ellernholzstr. 1-2, 17487 Greifswald, Germany

**Keywords:** Primary prevention, Cardiovascular risk, Total cholesterol, Blood pressure measurement

## Abstract

**Background:**

To compare the reproducibility in total cholesterol (TC), systolic blood pressure (BP), and the resulting Systematic COronary Risk Evaluation (SCORE) obtained by an in-office cardio-preventive screening program (SP) and a subsequent program performed in a clinical trial examination center (EP).

**Methods:**

A total of 307 individuals (60.3% female, mean age = 52.8 years) participated. According to TC and BP measurements at the SP and EP, three variables were created: the SCORE^SP^ = single BP reading at the SP, the SCORE^EP/BP-first^ = first BP reading at the EP, and the SCORE^EP/BP-mean^ = mean second/third BP reading at the EP. Differences in TC and BP were analyzed. Associations between age, sex and mean differences between the SCORE^SP^ and the SCORE^EP/BP-first^ (M1) and the SCORE^EP/BP-mean^ (M2) were analyzed using multivariable linear and quantile regression.

**Results:**

TC and BP values from the SP were significantly higher than those from the EP. Among individuals with a decreased SCORE value at the EP (compared to the SP), younger age was associated with a higher improvement in risk estimation compared with older age. Female sex was associated with higher risk improvement in the SCORE between the SP and the EP compared with male sex. Associations between both demographics and M1 (M2) achieved statistical significance at the 75.0th (50th) percentile.

**Conclusions:**

The reproducibility of results in cardiovascular risk prediction seems to be influenced by the accuracy of BP measurement. It seems that younger individuals and females are more likely to benefit from accuracy compared with older individuals and males.

**Electronic supplementary material:**

The online version of this article (10.1186/s12872-018-0823-3) contains supplementary material, which is available to authorized users.

## Background

European guidelines recommend the risk assessment of individuals free from cardiovascular disease (CVD) to be based on the Systematic COronary Risk Evaluation (SCORE) algorithm [1]. The SCORE determines the 10-year risk of a fatal atherosclerotic event using predictors such as age, sex, smoking status, total cholesterol (TC), and systolic blood pressure (BP) [1].

While age and sex can be determined with certainty, there is a degree of uncertainty in the determination of smoking status, TC and blood pressure values [2]. Measurement of TC and BP are subject to error based on their biological variation and methodological issues [3], and the consequences of these measurement issues on CVD risk prediction have been studied. A simulation study indicated that less of the intra-individual variability in the 10-year risk of fatal and non-fatal CVD events (Framingham algorithm) was explained by variation in TC, as most was explained by variation in blood pressure [2]. Further studies confirmed low intra-individual variability in TC up to 10 years [4, 5].

Whereas blood pressure measurement in clinical research is based on highly standardized readings according to established guidelines [4], blood pressure measurement in routine care rarely achieves this high level of accuracy [6, 7], resulting in higher readings compared to standardized measurements [8, 9].

Because the impact of intra-individual variability in TC and blood pressure levels are also related to the measurement standards of SCORE, the aim of this study was to evaluate the reproducibility of both cardiovascular parameters in a sample of individuals aged 40 and 65 years. In addition to the evidence mainly derived from simulation studies [2, 3], reproducibility was determined by comparing TC, BP, and the resulting SCORE values obtained by an in-office cardio-preventive screening program (SP) and a subsequent examination program (EP), performed in a clinical trial examination center. We report findings on the potential differences in TC, BP and the resulting SCORE according to three standardized blood pressure measurements, and we examined if sex and age are related to SCORE reproducibility.

## Methods

### Design and procedure

This analysis was part of a series of studies (June 2012 to December 2013) investigating different population groups in northeast Germany (general practice patients, job agency clients, and health insurance members) within a stepwise examination program addressing cardiovascular health [10, 11]. The first part of the study, an in-office SP, included a self-administered computerized assessment of cardiovascular risk factors. The opportunity to receive a single blood pressure measurement and non-fasting blood sample was offered. For the second part of the study, the EP, subjects without residence in the study area, with a history of cardiovascular events (myocardial infarction, stroke), vascular intervention, diabetes mellitus, and self-reported body mass index > 35 kg/m^2^ were excluded. The EP was conducted in the clinical trial examination center at the university hospital and included multiple standardized blood pressure measurements and a non-fasting blood sample.

### Selection of participants

The flow of participants has been described in more detail elsewhere [10, 11]. Although individuals aged 40 to 75 years were eligible for the study, the recruited job center clients were between 40 and 65 years old, because the maximum age to register at a job center is 65. Therefore, only participants between 40 and 65 years were considered. A total of 2614 individuals were eligible, and 930 (35.6%) participated in the computer-assisted assessment, which was the first part of the SP. A total of 568 individuals were eligible to participate in the EP, of whom 460 (80.9%) participated. Among them, all individuals with complete data regarding TC and BP at the SP and in the EP were included (*N* = 307).

#### Measures

Self-reported data on sex, age and current smoking status were collected within the SP. TC and BP were assessed during both, the SP and the EP. TC was determined from blood samples using standard methodology at the Institute of Clinical Chemistry at the University Medicine Greifswald. A single blood pressure reading of the right arm in the seated position was taken during the SP. During the EP, blood pressure measurement was performed according to a standardized protocol by a certified nurse [12]. The first reading was taken after a 5-min rest. In total, three readings of the right arm and one reading of the left arm were taken at 3-min intervals. For all blood pressure measurements, an Omron 705IT blood pressure monitor (Omron Corporation, Tokyo, Japan) was used.

Variables used for the SCORE, in addition to TC and BP included age, sex, and smoking status. Since the standard of blood pressure measurement might be a key component for the SCORE, three variables were built. The first variable (SCORE^SP^) included the result of TC and a single BP as the basis of the SP. The second variable and the third variable included TC and the first reading of BP (SCORE^EP/BP-first^) or the mean of the second and third readings of BP (SCORE^EP/BP-mean^) as the basis of the EP. The calculation of the SCORE was based on the equation scheme suggested by Conroy and colleagues [13].

#### Statistical analyses

Descriptive statistics were used to characterize the sample. Differences in TC and BP between participants in the SP and the EP were analyzed using linear mixed effect models with the individual as the random factor. All models were adjusted for sex, age, setting of recruitment and period between SP and EP (Mean = 24.1 days, SD = 24.8 days). Given that TC and BP measurements were viewed as the targets, two-way random effects models were used to estimate the Intraclass Correlation Coefficient (ICC, absolute agreement, average measures) with a 95% confidence interval across the two time points. We used the convention in which ICC values between 0.40 and 0.75 indicate fair to good correlation and values of 0.75 or greater indicate excellent correlation [14]. Estimates of the SCORE [1, 15] were calculated according to the three blood pressure measurement protocols. Pearson’s chi-square test was used to analyze if differences between SCORE^SP^ and SCORE^EP/ BP-first^ and between SCORE^SP^ and SCORE^EP/ BP-mean^ were statistically significant. Further, we performed multi-variable linear regression analyses, which estimated the average effect of age and sex on differences in SCORE (Outcome between SCORE^SP^ and SCORE^EP/BP-first^ (Model 1) and between SCORE^SP^ and SCORE^EP/ BP-mean^ (Model 2). Subsequently, differences in SCORE and covariates (age, sex) were also used in quantile regression for a more in-depth evaluation of effect size [16]. Compared with linear regression based on the conditional mean, quantile regression is more suitable in cases where the effect of covariates differs at different levels of the dependent variable. Thus, we tested whether potential associations of sex and age vary across the 2.5th to the 97.5th percentiles of individual SCORE differences. All regression models were adjusted for the setting of recruitment, duration between the SP and EP, and the SCORE value at SP. A *p* value < .05 was considered statistically significant. All analyses were carried out using Stata 14.1.

## Results

### Sociodemographic characteristics of the participants

The proportions of male individuals recruited via general practices (*n* = 154), from health insurance (*n* = 96), and from job agencies (*n* = 57) were 41.8%, 31.3 and 53.3%, respectively. The mean age was higher among individuals recruited from health insurance (56.6 ± 7.0) compared to individuals recruited via general practices (52.6 ± 7.3, *p* < 0.001) and job agencies (50.2 ± 5.8, *p* < 0.001). The prevalence of smoking was higher among individuals recruited from job agencies (58.7%) compared with those recruited from health insurance (15.7%, *p* < 0.001) and general practices (26.6%, *p* < 0.001).

### Total cholesterol, blood pressure and cardiovascular risk prediction using SCORE

The ICCs indicated an excellent agreement of TC and BP between SP and EP with one exception; the ICC for systolic blood pressure at the SP and the first reading in the EP was found to be fair to good (Table 1). However, analysis revealed that TC and BP values from the SP were significantly higher than those from the EP. Both findings were also seen for all three settings when analyzed separately (See Additional file 1: Table S1 and Additional file 2: Table S2).

**Table 1:** Differences in total cholesterol (mmol/l) and systolic blood pressure (mmHg) between the in-office cardio-preventive screening and the clinical examination program^a^

	In-office screening program	Clinical examination program^b^			Intraclass correlation		Clinical examination program^c^			Intraclass correlation	
	Mean (SE)	Mean (SE)	Mean difference (SE)	*p*-value	ICC	95%-CI	Mean(SE)	Mean difference^c^ (SE)	*p*-value	ICC	95%-CI
Total cholesterol	5.75 (0.63)	5.54 (0.58)	0.21 (0.03)	< .001	.91	.87–.94					
Systolic blood pressure	140.48 (1.00)	133.97 (0.99)	6.51 (0.82)	< .001	.60	.27–.75	128.63 (0.87)	11.86 (0.83)	< .001	. 91	.84–.95

*SE* standard error, *IC* intraclass correlation coefficient, *CI* confidence interval

^a^Adjusted for sex, age, setting of recruitment and duration between the in-office screening and the clinical examination program. The value of systolic blood pressure measurement corresponds to the first reading^b^, to the mean of the second and third reading^c^

Further, the calculation of the SCORE on the basis of measurements at the SP resulted in significantly higher proportions of individuals at moderate or high risk compared to calculation on the basis of measurements at the EP (Table 2).

**Table 2:** Proportions in Systematic COronary Risk Evaluation categories (*n* = 307) at the in-office cardio-preventive screening program and at the clinical examination program

	In-office screening program		Clinical examination program			
	SCORE^SP^		SCORE^EP/BP-first^^†^		SCORE^EP/BP-mean^^††^	
	n	%	n	%	n	%
Low risk						
SCORE < 1%	131	42.7	143	46.6	157	51.1
Moderate risk						
SCORE ≥ 1% and < 5%	154	50.2	151	49.2	143	46.6
High risk						
SCORE ≥ 5%	22	7.1	13	4.2	7	2.3

SCORE^SP^ was based on total cholesterol and the single reading of systolic blood pressure at the in-office screening program. SCORE^EP/BP-first^, (SCORE^EP/BP-mean^) were based on total cholesterol and the first reading (the mean of the second and third reading) of systolic blood pressure at the clinical examination program

^†^Pearson chi^2^ = 330.996; *p* < .001, comparison between SCORE^SP^ and SCORE^EP/BP-first^

^††^Pearson chi^2^ = 159.592; *p* < .001, comparison between SCORE^SP^ and SCORE^EP/BP-mean^

Table 3 gives a comparison of the coefficients obtained using multivariable linear regression and quantile regression. Multivariable linear regression analysis revealed that sex and age were not associated with mean differences between SCORE^SP^ and SCORE^EP/BP-first^ (Model 1) and between SCORE^SP^ and SCORE^EP/BP-mean^ (Model 2). According to the quantile regression analysis, age was negatively associated with the 50.0th percentile (Model 2 only) and with the 75.0th and the 90.0th percentiles (both Models) of mean differences in SCORE. Female sex was positively associated with the 50.0th percentile (Model 2 only) and with the 75.0th and the 90.0th percentiles (both Models) of mean differences in SCORE.

**Table 3:** Associations between age, sex and the mean differences in Systematic COronary Risk Evaluation between the in-office cardio-preventive screening program and clinical examination program^a^

	Linear regression	Quantile regression				
		q10	q25	q50	q75	q90
	*b* [95% CI]	*b* [95% CI]	*b* [95% CI]	*b* [95% CI]	*b* [95% CI]	*b* [95% CI]
Model 1: Difference between SCORE^SP^ and SCORE^EP/BP-first^						
Age	−0.012 [−0.048, 0.025]	−0.013 [− 0.043, 0.016]	−0.009 [− 0.026, 0.008]	−0.013 [− 0.029, 0.003]	**−0.021 [− 0.033, − 0.009]**	**−0.011 [− 0.020, − 0.002]**
Sex	0.025 [− 0.388, 0.438]	0.045 [− 0.330, 0.420]	0.084 [− 0.127, 0.295]	0.111 [− 0.094, 0.317]	**0.204 [0.051, 0.356]**	**0.153 [0.039, 0.268]**
Model 2: Difference between SCORE^SP^ and SCORE^EP/BP-mean^						
Age	− 0.003 [− 0.041, 0.035]	−0.003 [− 0.014, 0.008]	−0.004 [− 0.024, 0.015]	**−0.014 [− 0.262, − 0.002]**	**−0.017 [− 0.028, − 0.006]**	**−0.022 [− 0.030, − 0.129]**
Sex	0.075 [− 0.349, 0.499]	0.046 [0.103, 0.194]	0.132 [− 0.121, 0.385]	**0.176 [0.020, 0.332]**	**0.202 [0.059, 0.344]**	**0.247 [0.135, 0.359]**

Bold coefficients indicate significant effects

*q* quantile, *b* coefficient, *CI* confidence interval

^a^Analyses were adjusted for setting of recruitment, duration between cardio-preventive screening and clinical examination program, and the SCORE value at the in-office screening program. SCORE^SP^ was based on total cholesterol and the single reading of systolic blood pressure at the in-office screening program. SCORE^EP/BP-first^ (SCORE^EP/BP-mean^) were based on total cholesterol and the first reading (the mean of the second and third reading) of systolic blood pressure at clinical examination program

**Fig. 1:**
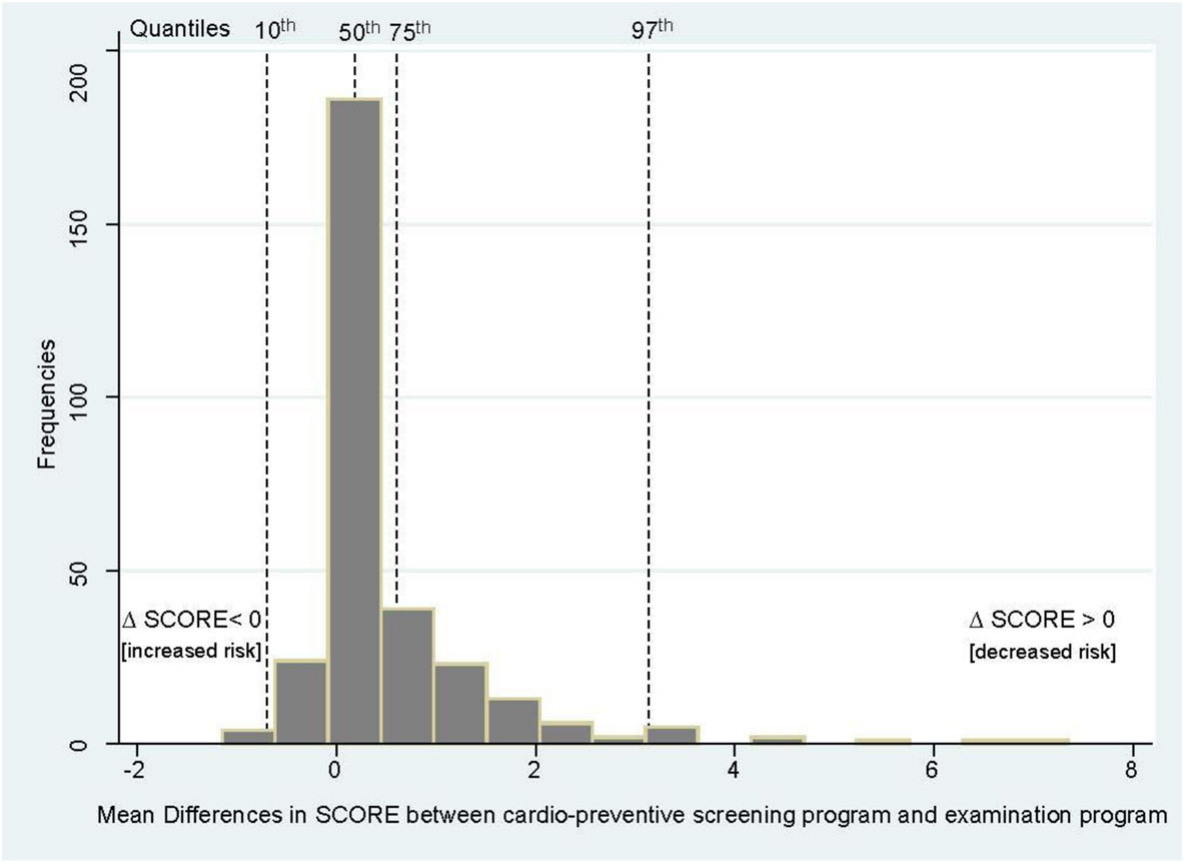
Distribution of mean differences in SCORE^1^ between the in-office screening and the clinical examination program (2.5–99.0th percentile). Legends ^1^ ∆ SCORE = SCORE^SP^ (based on total cholesterol and the single reading of systolic blood pressure at the in-office screening program) – SCORE ^SP/BPmean^ (based on total cholesterol and the mean of the second and third reading of systolic blood pressure at clinical examination program)

The distribution of mean differences in the SCORE (∆ SCORE) between SCORE^SP^ and SCORE^EP/BP-mean^ in relation to the number of individuals is presented in Fig. 1. While ∆ SCORE < 0 determined an increased SCORE value, ∆ SCORE > 0 determined a decreased SCORE value, both at the EP and in comparison to the SP.

### The relation of sex and age to SCORE reproducibility

**Fig. 2:**
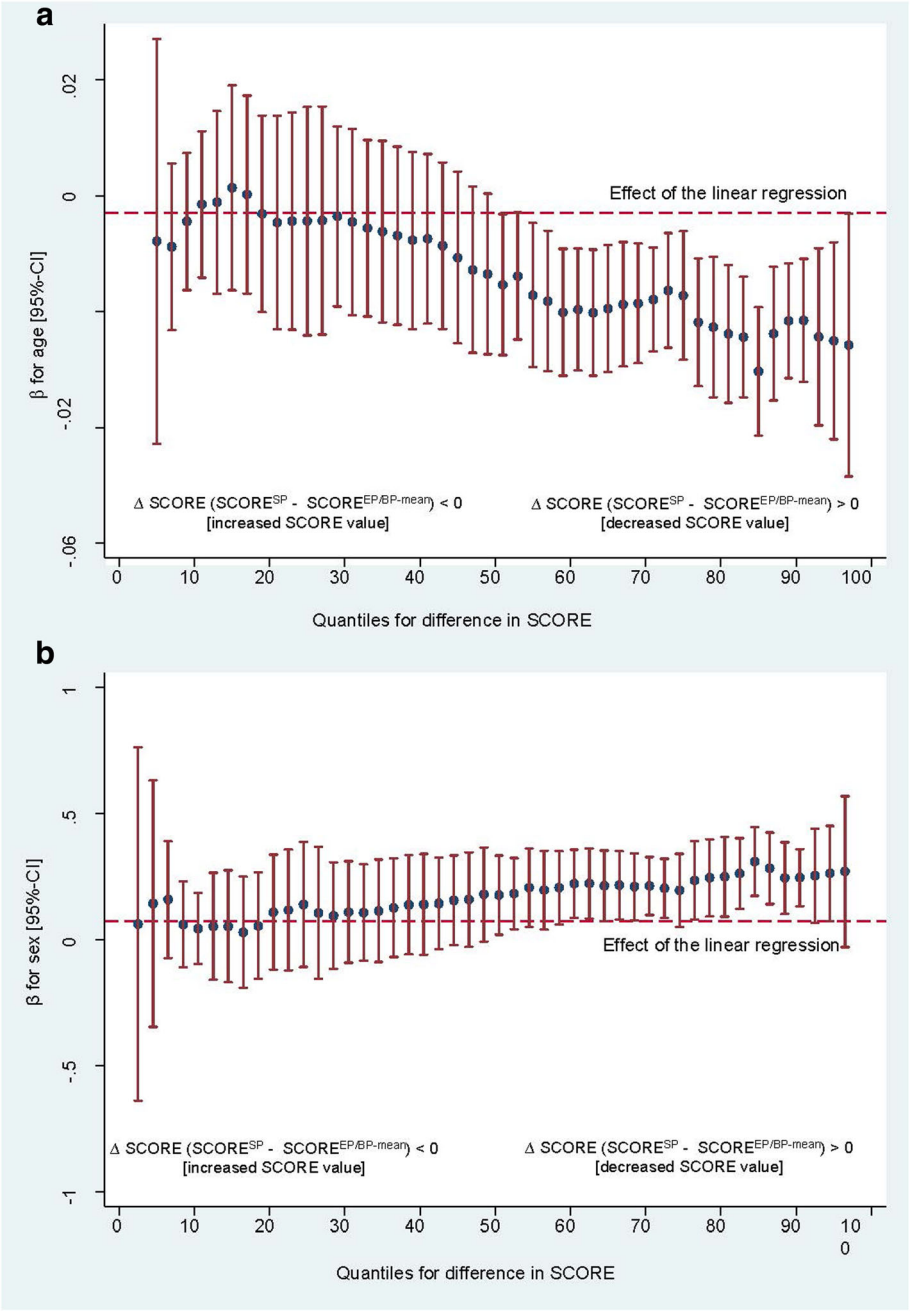
Variation in regression coefficients of age (**a**) and sex (**b**) on mean SCORE difference between the in-office screening and clinical examination program (2.5th - 97.5th percentile)^1^. Legends ^1^ SCORE^SP^ was based on total cholesterol and the single reading of systolic blood pressure at the in-office screening program). SCORE ^SP/BPmean^ was based on total cholesterol and the mean of the second and third reading of systolic blood pressure at clinical examination program

The variation in regression coefficients for age and sex according to the 2.5th to the 97.0th percentiles in Model 2 are presented in Fig. 2a and b. As shown in Fig. 2a, the inverse association between the mean SCORE difference and age became stronger and statistically significant only in the higher percentiles of the outcome variable, e.g., at the 97.0th percentile (Mean SCORE difference = 3.141, *b* = − 0.026, 95%-CI: -0.048, − 0.0038), where the age effect was more pronounced compared to the 50.0th percentile (Mean SCORE difference = 0.182, *b* = − 0.014. 95%-CI: -0.262, − 0.002). This finding means that in individuals with lower risk estimations at the EP, younger individuals have a higher risk estimation improvement compared to older individuals due to the highly-standardized measurement regime at the EP.

As shown in Fig. 2b the positive association between female sex and mean differences in SCORE strengthened in the higher percentiles again and achieved statistical significance at the 50.0th percentile. This finding means that, in individuals with lower risk estimation at the EP, females have a higher risk estimation improvement compared to males.

## Discussion

The three main findings of our study are as follows: (1) The results of TC and BP measurement varied between the SP and EP with lower values derived from measurements at EP. (2) The proportions of individuals at moderate or high risk according to the SCORE at the EP were lower compared to the proportions at the SP. (3) The strength of the association between age, sex and the mean differences in the SCORE between the SP and the EP varied substantially across the distribution of our outcome, which was the mean difference in the SCORE.

The impact of combined risk factors for CVD risk estimation is emphasized in the guidelines recently published by the European Society of Cardiology [1]. While age, sex and smoking status were stable due to the short time period between the SP and the EP, our data indicated a relative difference of 3.6% (0.21 mmol/l) in TC between both measurements. Observed changes in TC measurements are often due to errors related to processing, storage and laboratory analyses and biological variability [17] and/or a short-term biological variability up to 7.0% [18]. However, the mean of BP (140.5 mmHg) obtained by a single measurement at the SP was found to be decreased by 4.6% (6.5 mmHg) at the EP when adhering to a resting time of 5 min prior to the first measurement and by 11.9% (8.4 mmHg) when calculating the mean of the second and third measurements. Given our results and that the majority of studies used guidelines proposed repeated BP measurements, taking a single measurement without resting period should be discouraged in clinical routine care. However, increasing the accuracy of BP measurement in clinical routine care seems to be a challenge because 77% of physicians took BP reading without any resting time [19].

The error introduced by low standardization of blood pressure measurement at the SP in addition to biological variation [20] affects the second finding of our study, the variability on cardiovascular risk prediction using SCORE. Imprecision in calculation is important, especially if the SCORE is close to the threshold of high risk, as subjects may benefit from intensive counseling and/or drug treatment. However, similar to the results of a simulation study, moderate- and high-risk individuals in our study were more likely subjected to errors in the SCORE than low-risk participants [3].

The third main finding of this study is related to the differential impact of age and sex across the distribution of mean differences in the SCORE between the SP and the EP. Our results add to the literature by showing that neither sex nor age were found to be associated with mean differences in the SCORE across the whole distribution.

When linear multivariable regression analyses were employed, the results failed to capture the differential association found when using quantile regression. The mean differences in the SCORE between the SP and the EP (SCORE ^EP/SPB-mean^) were 0.182 at the 50.0th percentile and 3.141 at the 97.0th percentile. Among individuals with a decreased SCORE value at the EP (compared to the SP), younger age was associated with a higher decrease and therefore with a higher improvement in risk estimation compared with older age. The association was strongest at the higher percentiles of the outcome. Furthermore, our results suggest that female sex is associated with higher risk improvement in the SCORE between the SP and the EP compared with male sex. However, the association was found to be significant only for percentiles ≥50.0th of the outcome. It seems that younger individuals and females are more dependent on the measurement situation (unscheduled in-office examination program vs. scheduled clinical trial center examination program) and/or the measurement regimes. The present study linked CVD risk prediction data assessed close to routine practice to those assessed via highly standardized measurement in a clinical trial examination center. Thus, our results extend the evidence regarding the effects of inter-individual variability in TC and BP on CVD risk estimation as shown in simulation studies [2, 3].

However, our study is subject to at least four limitations. First, all findings are based on a relatively small sample of individuals aged between 40 and 65 years. Second, individuals eligible for the EP were only those without pre-existing diabetes and cardiovascular disease, resulting in a reduced number of individuals categorized as high risk according to the SCORE. Furthermore, smokers were less likely to participate in the EP [10], which might have contributed to the low number of high-risk individuals. Third, other potential confounding factors including those related to modifiable risk factors (e. g. physical activity) were not investigated in the present study.

## Conclusions

The reproducibility of results in cardiovascular risk prediction using SCORE seems to be influenced particularly by the accuracy of BP measurement. Younger individuals and females are more likely to benefit from a resting period prior to the first BP reading and in addition from two more BP readings, taken at 3-min intervals, compared with older individuals and males.

## Additional files

Additional file 1: **Table S1.** Differences in total cholesterol (mmol/l) between the cardio-preventive screening and the clinical examination program. Results from total cholesterol measurement at the cardio-preventive screening program, and at clinical examination program, analyzed separately for general practice patients, job agency clients, and health insurance members. (DOCX 14 kb)

Additional file 2: **Table S2.** Differences in systolic blood pressure (mmHg) and total cholesterol (mmol/l) between the cardio-preventive screening and the clinical examination program according to the setting of recruitment. Results from blood pressure measurement a the the cardio-preventive screening program, and at clinical examination program, analyzed separately for general practice patients, job agency clients, and health insurance members. (DOCX 20 kb)

## Abbreviations

b: Coefficient
BP: Systolic blood pressure
CI: Confidence interval
EP: Examination program
ICC: Intraclass correlation coefficient
q: Quantile
SCORE: Systematic COronary Risk Evaluation
SE: Standard error
SP: Screening program
TC: Total cholesterol
