# Supplementary material for: Effect of blood pressure and total cholesterol measurement on risk prediction using the Systematic COronary Risk Evaluation (SCORE)
Source: BMC Cardiovasc Disord. 2018 May 4;18:84. doi: 10.1186/s12872-018-0823-3 (PMC5935918; doi:10.1186/s12872-018-0823-3)
Supplement: Supplementary file 1 — Table S1. Differences in total cholesterol (mmol/l) between the cardio-preventive screening and the clinical examination program. Results from total cholesterol measurement at the cardio-preventive screening program, and at clinical examination program, analyzed separately for general practice patients, job agency clients, and health insurance members. (DOCX 14 kb) [file 12872_2018_823_MOESM1_ESM.docx]

| Table S1 Differences in total cholesterol (mmol/l) between the cardio-preventive screening and the clinical examination program ^1^ | | | | | | |
| --- | --- | --- | --- | --- | --- | --- |
|  | Screening program | Clinical examination program | | | Intraclass correlation | |
|  | Mean (SE) | Mean (SE) | Mean difference^1^ | p-value | ICC | 95% CI |
| General practices | | | | |  |  |
|  | 5.73 (0.09) | 5.59 (0.08) | 0.14 (0.05) | 0.018 | .90 | .87 - .93 |
| Job agency | | | | |  |  |
|  | 5.90 (0.16) | 5.60 (0.14) | 0.30 (0.77) | 0.001 | .92 | .83 - .96 |
| Health insurance | | | | |  |  |
|  | 5.71 (0.10) | 5.42 (0.10) | 0.29 ( 0.05) | <.001 | .92 | .85 - .95 |

^1^ Analysis was adjusted for age, sex, setting of recruitment and duration between the screening and the clinical examination program. Abbreviations: ICC = Intraclass correlation coefficient, CI = Confidence interval
