# Supplementary material for: Effect of blood pressure and total cholesterol measurement on risk prediction using the Systematic COronary Risk Evaluation (SCORE)
Source: BMC Cardiovasc Disord. 2018 May 4;18:84. doi: 10.1186/s12872-018-0823-3 (PMC5935918; doi:10.1186/s12872-018-0823-3)
Supplement: Supplementary file 2 — Table S2. Differences in systolic blood pressure (mmHg) and total cholesterol (mmol/l) between the cardio-preventive screening and the clinical examination program according to the setting of recruitment. Results from blood pressure measurement a the the cardio-preventive screening program, and at clinical examination program, analyzed separately for general practice patients, job agency clients, and health insurance members. (DOCX 20 kb) [file 12872_2018_823_MOESM2_ESM.docx]

| Table S2 Differences in systolic blood pressure (mmHg) and total cholesterol (mmol/l) between the cardio-preventive screening and the clinical examination program according to the setting of recruitment ^1^ | | | | | | | |
| --- | --- | --- | --- | --- | --- | --- | --- |
|  | Screening program ^2^ | Clinical examination program ^3^ Intraclass correlation | | | Clinical examination program^4^ Intraclass correlation | | |
|  | Mean (SE) | Mean (SE) | Mean difference (SE) | p-value ICC 95% CI | Mean (SE) | Mean difference (SE) | p-value ICC 95% CI |
| Systolic blood pressure | |  |  |  |  |  |  |
| General practices | 140.76  (1.27) | 134.84  (1.36) | 5.92  (1.13) | <.001 .79 .67 - .86 | 129.36  (1.14) | 11.40  (1.11) | <.001 .69 .21 - .84 |
| Job agency | 144.46  (2.60) | 133.37  (2.10) | 11.09  (2.24) | <.001 .65 .26 - .82 | 129.24  (1.88) | 15.22  (2.22) | <.001 .56 -.02 - .79 |
| Health insurance | 137.67  (1.86) | 132.93  (1.93) | 4.74  (1.45) | <.001 .86 .78 - .91 | 127.06  (1.73) | 10.60  (1.45) | <.001 .79 .41 - .90 |
| Total cholesterol |  |  |  |  |  |  |  |
| General practices | 5.73  (0.09) | 5.59  (0.08) | 0.14  (0.05) | 0.018 .90 .87 - .93 |  |  |  |
| Job agency | 5.90  (0.16) | 5.60  (0.14) | 0.30  (0.77) | 0.001 .92 .83 - .96 |  |  |  |
| Health insurance | 5.71  (0.10) | 5.42  (0.10) | 0.29  (0.05) | <.001 .92 .85 - .95 |  |  |  |

^1^ Adjusted for age, sex, setting of recruitment and duration between the cardio-preventive screening and the clinical examination program. The calculation of the systolic blood pressure value were based on ^1^ the single reading at the cardio-preventive screening program, ^2^ the first reading, and ^3^ the mean of the second and third reading at the clinical examination program.
